# (*E*)-2-(2-Formyl­phen­oxy­meth­yl)-3-phenyl­prop-2-ene­nitrile

**DOI:** 10.1107/S1600536811025670

**Published:** 2011-07-09

**Authors:** K. Swaminathan, K. Sethusankar, G. Murugan, M. Bakthadoss

**Affiliations:** a Department of Physics, RKM Vivekananda College (Autonomous), Chennai 600 004, India; b Department of Organic Chemistry, University of Madras, Maraimalai Campus, Chennai 600 025, India

## Abstract

In the title compound, C_17_H_13_NO_2_, the dihedral angle between the benzene and the phenyl ring is 65.92 (7)°. The carbonitrile side chain is almost linear, the C—C—N angle being 175.55 (14)°. The crystal structure is stabilized by inter­molecular C—H⋯O inter­actions.

## Related literature

For background to the synthesis, see: Bakthadoss & Murugan (2010 ▶). For a related structure, see: Jasinski *et al.* (2011 ▶).
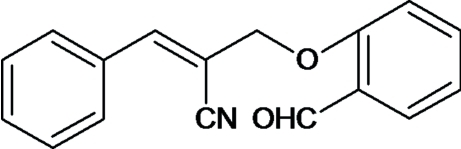


## Experimental

### 

#### Crystal data

C_17_H_13_NO_2_
*M* *_r_* = 263.28
Triclinic, 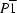

*a* = 8.0157 (4) Å
*b* = 9.2589 (4) Å
*c* = 10.2348 (5) Å
α = 68.283 (2)°
β = 73.432 (2)°
γ = 79.804 (2)°
*V* = 674.20 (6) Å^3^
*Z* = 2
Mo *K*α radiation
μ = 0.09 mm^−1^
*T* = 293 K
0.30 × 0.25 × 0.25 mm

#### Data collection

Bruker Kappa APEXII CCD diffractometer
12600 measured reflections
2628 independent reflections
2162 reflections with *I* > 2σ(*I*)
*R*_int_ = 0.023

#### Refinement

*R*[*F* ^2^ > 2σ(*F* ^2^)] = 0.037
*wR*(*F* ^2^) = 0.102
*S* = 1.03
2628 reflections
181 parameters
H-atom parameters constrained
Δρ_max_ = 0.12 e Å^−3^
Δρ_min_ = −0.17 e Å^−3^

### 

Data collection: *APEX2* (Bruker, 2004 ▶); cell refinement: *SAINT* (Bruker, 2004 ▶); data reduction: *SAINT*; program(s) used to solve structure: *SHELXS97* (Sheldrick, 2008 ▶); program(s) used to refine structure: *SHELXL97* (Sheldrick, 2008 ▶); molecular graphics: *ORTEP-3* (Farrugia, 1997 ▶) and *Mercury* (Macrae *et al.*, 2006 ▶); software used to prepare material for publication: *SHELXL97* and *PLATON* (Spek, 2009 ▶).

## Supplementary Material

Crystal structure: contains datablock(s) global, I. DOI: 10.1107/S1600536811025670/pv2417sup1.cif

Structure factors: contains datablock(s) I. DOI: 10.1107/S1600536811025670/pv2417Isup2.hkl

Supplementary material file. DOI: 10.1107/S1600536811025670/pv2417Isup3.cml

Additional supplementary materials:  crystallographic information; 3D view; checkCIF report

## Figures and Tables

**Table 1:** Hydrogen-bond geometry (Å, °)

*D*—H⋯*A*	*D*—H	H⋯*A*	*D*⋯*A*	*D*—H⋯*A*
C12—H12⋯O2^i^	0.93	2.48	3.2675 (17)	143

Symmetry code: (i) 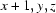.

**Fig. 1.**
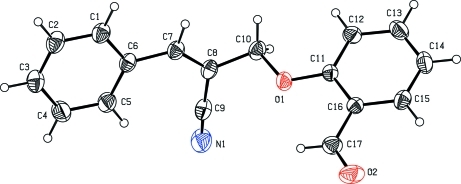
The molecular structure of the title compound with the atom numbering scheme. Displacement ellipsoids are drawn at 30% probability level.

**Fig. 2.**
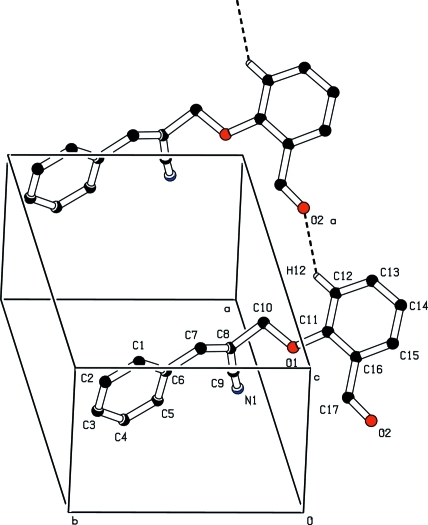
Packing arrangement of the title compound; C—H···O intermolecular interactions are indicated by dashed lines.

## supplementary crystallographic information

### Comment

The title compound is a stereodefined trisubstituted olefin, synthesized from the corresponding bromoderivative of Baylis-Hillman adduct with salicylaldehyde *via* simple SN2 reaction in good yields. This *o*-salicyladehyde derivative is an important precursor for many heterocyclic frameworks (Bakthadoss *et al.*, 2010).

The title compund comprises a phenyl ring connected to a benzaldehyde through a chain of acrylonitrile and methoxymethyl groups (Fig. 1). The phenyl rings (C1—C6) and  (C11—C16) form  dihedral angles of 6.46 (8) and 71.66 (6)°, respectively, with the plane formed by the atoms (N1/C8—C10); the dihedral angle between the two phenyl rings is 65.92 (7)°. The deviation of the atom O2 in the aldehyde group, from the mean plane of the phenyl ring (C11—C16) is 0.0709 (12)Å. The bond angle around C9, in the chain of atoms N1/C9/C8, is 175.55 (14)° and thus the carbonitrile side chain is almost linear. The crystal packing is stabilized by intermolecular C—H···O interactions (Tab. 1 and Fig. 2).

### Experimental

A solution of salicylaldehyde (1.0 mmol, 0.122 g) and potassium carbonate (2.0 mmol, 0.2293 g) in acetonitrile solvent (5 ml) was stirred for 15 minute at room temperature. To this solution, (*E*)-2-(bromomethyl)-3-phenylacrylonitrile (1.2 mmol, 0.27 g) was added dropwise. After the completion of the reaction, as indicated by TLC, acetonitrile was evaporated. EtOAc (15 ml) and water (15 ml) were added to the crude mass. The organic layer was dried over anhydrous sodium sulfate. Removal of solvent led to the crude product, which was purified through pad of silica gel (100–200 mesh) using ethylacetate and hexanes(1:9) as solvents. The pure title compound was obtained as a colourless solid (0.22 g, 84% yield). Recrystallization was carried out using methanol as solvent.

### Refinement

Hydrogen atoms were placed in calculated positions with C—H = 0.93 - 0.97 Å, and refined in riding model with fixed isotropic displacement parameters: *U*_iso_(H) = 1.2 *U*_eq_(C).

### Crystal data

**Table:**

C_17_H_13_NO_2_	*Z* = 2
*M**_r_* = 263.28	*F*(000) = 276
Triclinic, *P*1	*D*_x_ = 1.297 Mg m^−^^3^
Hall symbol: -P 1	Mo *K*α radiation, λ = 0.71073 Å
*a* = 8.0157 (4) Å	Cell parameters from 2628 reflections
*b* = 9.2589 (4) Å	θ = 1.0–26.0°
*c* = 10.2348 (5) Å	µ = 0.09 mm^−^^1^
α = 68.283 (2)°	*T* = 293 K
β = 73.432 (2)°	Block, colourless
γ = 79.804 (2)°	0.30 × 0.25 × 0.25 mm
*V* = 674.20 (6) Å^3^	

### Data collection

**Table:**

Bruker Kappa APEXII CCD diffractometer	2162 reflections with *I* > 2σ(*I*)
Radiation source: fine-focus sealed tube	*R*_int_ = 0.023
graphite	θ_max_ = 26.0°, θ_min_ = 2.6°
ω scans	*h* = −9→9
12600 measured reflections	*k* = −11→11
2628 independent reflections	*l* = −12→12

### Refinement

**Table:**

Refinement on *F*^2^	Primary atom site location: structure-invariant direct methods
Least-squares matrix: full	Secondary atom site location: difference Fourier map
*R*[*F*^2^ > 2σ(*F*^2^)] = 0.037	Hydrogen site location: inferred from neighbouring sites
*wR*(*F*^2^) = 0.102	H-atom parameters constrained
*S* = 1.03	*w* = 1/[σ^2^(*F*_o_^2^) + (0.0528*P*)^2^ + 0.0828*P*] where *P* = (*F*_o_^2^ + 2*F*_c_^2^)/3
2628 reflections	(Δ/σ)_max_ < 0.001
181 parameters	Δρ_max_ = 0.12 e Å^−^^3^
0 restraints	Δρ_min_ = −0.17 e Å^−^^3^

### Special details

**Table:**

Geometry. All e.s.d.'s (except the e.s.d. in the dihedral angle between two l.s. planes) are estimated using the full covariance matrix. The cell e.s.d.'s are taken into account individually in the estimation of e.s.d.'s in distances, angles and torsion angles; correlations between e.s.d.'s in cell parameters are only used when they are defined by crystal symmetry. An approximate (isotropic) treatment of cell e.s.d.'s is used for estimating e.s.d.'s involving l.s. planes.
Refinement. Refinement of *F*^2^ against ALL reflections. The weighted *R*-factor *wR* and goodness of fit *S* are based on *F*^2^, conventional *R*-factors *R* are based on *F*, with *F* set to zero for negative *F*^2^. The threshold expression of *F*^2^ > σ(*F*^2^) is used only for calculating *R*-factors(gt) *etc*. and is not relevant to the choice of reflections for refinement. *R*-factors based on *F*^2^ are statistically about twice as large as those based on *F*, and *R*- factors based on ALL data will be even larger.

### Fractional atomic coordinates and isotropic or equivalent isotropic displacement parameters (Å^2^)

**Table:**

	*x*	*y*	*z*	*U*_iso_*/*U*_eq_	
C1	0.26890 (18)	0.64113 (15)	0.64335 (14)	0.0532 (3)	
H1	0.3008	0.6132	0.7305	0.064*	
C2	0.2241 (2)	0.79493 (15)	0.57315 (15)	0.0595 (4)	
H2	0.2270	0.8700	0.6125	0.071*	
C3	0.17519 (19)	0.83824 (15)	0.44570 (16)	0.0602 (4)	
H3	0.1450	0.9425	0.3981	0.072*	
C4	0.1710 (2)	0.72668 (16)	0.38826 (16)	0.0648 (4)	
H4	0.1372	0.7556	0.3017	0.078*	
C5	0.21649 (18)	0.57237 (15)	0.45793 (15)	0.0570 (3)	
H5	0.2129	0.4980	0.4180	0.068*	
C6	0.26761 (15)	0.52628 (13)	0.58692 (13)	0.0449 (3)	
C7	0.31669 (16)	0.36612 (13)	0.66887 (13)	0.0468 (3)	
H7	0.3373	0.3542	0.7574	0.056*	
C8	0.33774 (15)	0.23310 (13)	0.64123 (13)	0.0462 (3)	
C9	0.31738 (18)	0.22202 (14)	0.51102 (15)	0.0543 (3)	
C10	0.38224 (15)	0.08068 (13)	0.74939 (14)	0.0486 (3)	
H10A	0.4728	0.0193	0.7021	0.058*	
H10B	0.4233	0.0975	0.8227	0.058*	
C11	0.23087 (14)	−0.14432 (12)	0.91208 (12)	0.0385 (3)	
C12	0.37770 (16)	−0.21887 (14)	0.96098 (14)	0.0496 (3)	
H12	0.4804	−0.1690	0.9277	0.059*	
C13	0.37016 (19)	−0.36785 (15)	1.05952 (15)	0.0582 (4)	
H13	0.4686	−0.4175	1.0935	0.070*	
C14	0.2212 (2)	−0.44558 (15)	1.10927 (14)	0.0586 (4)	
H14	0.2196	−0.5474	1.1741	0.070*	
C15	0.07545 (17)	−0.37049 (14)	1.06170 (13)	0.0497 (3)	
H15	−0.0262	−0.4218	1.0954	0.060*	
C16	0.07657 (14)	−0.21887 (13)	0.96393 (11)	0.0395 (3)	
C17	−0.08123 (16)	−0.14054 (16)	0.91668 (14)	0.0534 (3)	
H17	−0.0751	−0.0399	0.8497	0.064*	
N1	0.3060 (2)	0.20353 (14)	0.40992 (15)	0.0774 (4)	
O1	0.22458 (10)	0.00245 (9)	0.81342 (9)	0.0484 (2)	
O2	−0.21885 (12)	−0.19734 (13)	0.95850 (12)	0.0737 (3)	

### Atomic displacement parameters (Å^2^)

**Table:**

	*U*^11^	*U*^22^	*U*^33^	*U*^12^	*U*^13^	*U*^23^
C1	0.0663 (8)	0.0489 (7)	0.0454 (7)	−0.0121 (6)	−0.0079 (6)	−0.0176 (6)
C2	0.0776 (10)	0.0449 (7)	0.0588 (8)	−0.0084 (6)	−0.0097 (7)	−0.0244 (6)
C3	0.0708 (9)	0.0423 (7)	0.0632 (9)	0.0010 (6)	−0.0138 (7)	−0.0173 (6)
C4	0.0848 (11)	0.0519 (8)	0.0624 (9)	0.0057 (7)	−0.0312 (8)	−0.0195 (7)
C5	0.0736 (9)	0.0456 (7)	0.0589 (8)	−0.0028 (6)	−0.0204 (7)	−0.0230 (6)
C6	0.0463 (7)	0.0423 (6)	0.0428 (6)	−0.0110 (5)	−0.0012 (5)	−0.0145 (5)
C7	0.0499 (7)	0.0448 (6)	0.0421 (6)	−0.0128 (5)	−0.0041 (5)	−0.0117 (5)
C8	0.0454 (7)	0.0422 (6)	0.0461 (7)	−0.0110 (5)	−0.0023 (5)	−0.0124 (5)
C9	0.0643 (8)	0.0384 (6)	0.0577 (8)	−0.0074 (6)	−0.0094 (6)	−0.0158 (6)
C10	0.0411 (7)	0.0437 (6)	0.0548 (7)	−0.0100 (5)	−0.0047 (5)	−0.0119 (5)
C11	0.0407 (6)	0.0357 (5)	0.0382 (6)	−0.0023 (4)	−0.0092 (5)	−0.0121 (5)
C12	0.0421 (7)	0.0504 (7)	0.0583 (8)	0.0000 (5)	−0.0167 (6)	−0.0189 (6)
C13	0.0644 (9)	0.0514 (7)	0.0619 (8)	0.0136 (6)	−0.0308 (7)	−0.0193 (6)
C14	0.0839 (10)	0.0380 (6)	0.0495 (7)	0.0008 (6)	−0.0211 (7)	−0.0087 (5)
C15	0.0609 (8)	0.0451 (6)	0.0419 (6)	−0.0154 (6)	−0.0047 (6)	−0.0139 (5)
C16	0.0416 (6)	0.0421 (6)	0.0358 (6)	−0.0063 (5)	−0.0073 (5)	−0.0145 (5)
C17	0.0436 (7)	0.0618 (8)	0.0535 (7)	−0.0085 (6)	−0.0120 (6)	−0.0159 (6)
N1	0.1142 (12)	0.0570 (7)	0.0704 (9)	−0.0038 (7)	−0.0291 (8)	−0.0284 (7)
O1	0.0401 (5)	0.0395 (4)	0.0557 (5)	−0.0078 (3)	−0.0119 (4)	−0.0026 (4)
O2	0.0447 (6)	0.1005 (8)	0.0789 (7)	−0.0212 (5)	−0.0146 (5)	−0.0272 (6)

### Geometric parameters (Å, °)

**Table:**

C1—C2	1.3719 (18)	C10—O1	1.4324 (13)
C1—C6	1.3879 (17)	C10—H10A	0.9700
C1—H1	0.9300	C10—H10B	0.9700
C2—C3	1.3656 (19)	C11—O1	1.3631 (13)
C2—H2	0.9300	C11—C12	1.3802 (16)
C3—C4	1.3735 (19)	C11—C16	1.3945 (15)
C3—H3	0.9300	C12—C13	1.3743 (18)
C4—C5	1.3758 (18)	C12—H12	0.9300
C4—H4	0.9300	C13—C14	1.374 (2)
C5—C6	1.3865 (17)	C13—H13	0.9300
C5—H5	0.9300	C14—C15	1.3684 (19)
C6—C7	1.4555 (17)	C14—H14	0.9300
C7—C8	1.3351 (16)	C15—C16	1.3900 (16)
C7—H7	0.9300	C15—H15	0.9300
C8—C9	1.4298 (19)	C16—C17	1.4575 (17)
C8—C10	1.4950 (17)	C17—O2	1.2026 (15)
C9—N1	1.1397 (17)	C17—H17	0.9300
			
C2—C1—C6	121.36 (12)	C8—C10—H10A	110.5
C2—C1—H1	119.3	O1—C10—H10B	110.5
C6—C1—H1	119.3	C8—C10—H10B	110.5
C3—C2—C1	120.28 (12)	H10A—C10—H10B	108.7
C3—C2—H2	119.9	O1—C11—C12	123.92 (10)
C1—C2—H2	119.9	O1—C11—C16	115.88 (9)
C2—C3—C4	119.54 (12)	C12—C11—C16	120.20 (10)
C2—C3—H3	120.2	C13—C12—C11	119.10 (12)
C4—C3—H3	120.2	C13—C12—H12	120.4
C3—C4—C5	120.40 (13)	C11—C12—H12	120.4
C3—C4—H4	119.8	C14—C13—C12	121.87 (12)
C5—C4—H4	119.8	C14—C13—H13	119.1
C4—C5—C6	120.90 (12)	C12—C13—H13	119.1
C4—C5—H5	119.6	C15—C14—C13	118.78 (12)
C6—C5—H5	119.6	C15—C14—H14	120.6
C5—C6—C1	117.51 (11)	C13—C14—H14	120.6
C5—C6—C7	124.85 (11)	C14—C15—C16	121.20 (12)
C1—C6—C7	117.62 (11)	C14—C15—H15	119.4
C8—C7—C6	132.27 (12)	C16—C15—H15	119.4
C8—C7—H7	113.9	C15—C16—C11	118.81 (11)
C6—C7—H7	113.9	C15—C16—C17	120.15 (11)
C7—C8—C9	124.29 (11)	C11—C16—C17	121.04 (10)
C7—C8—C10	121.16 (11)	O2—C17—C16	124.38 (12)
C9—C8—C10	114.54 (10)	O2—C17—H17	117.8
N1—C9—C8	175.55 (14)	C16—C17—H17	117.8
O1—C10—C8	106.15 (9)	C11—O1—C10	118.29 (9)
O1—C10—H10A	110.5		
			
C6—C1—C2—C3	0.6 (2)	C16—C11—C12—C13	0.92 (18)
C1—C2—C3—C4	0.1 (2)	C11—C12—C13—C14	0.9 (2)
C2—C3—C4—C5	−0.3 (2)	C12—C13—C14—C15	−1.7 (2)
C3—C4—C5—C6	−0.1 (2)	C13—C14—C15—C16	0.54 (19)
C4—C5—C6—C1	0.8 (2)	C14—C15—C16—C11	1.25 (17)
C4—C5—C6—C7	179.50 (13)	C14—C15—C16—C17	−179.03 (11)
C2—C1—C6—C5	−1.04 (19)	O1—C11—C16—C15	178.18 (10)
C2—C1—C6—C7	−179.87 (11)	C12—C11—C16—C15	−1.98 (16)
C5—C6—C7—C8	5.5 (2)	O1—C11—C16—C17	−1.54 (16)
C1—C6—C7—C8	−175.73 (13)	C12—C11—C16—C17	178.30 (11)
C6—C7—C8—C9	1.5 (2)	C15—C16—C17—O2	1.27 (19)
C6—C7—C8—C10	−177.29 (12)	C11—C16—C17—O2	−179.01 (12)
C7—C8—C10—O1	104.09 (13)	C12—C11—O1—C10	4.17 (16)
C9—C8—C10—O1	−74.78 (13)	C16—C11—O1—C10	−175.99 (9)
O1—C11—C12—C13	−179.25 (11)	C8—C10—O1—C11	176.89 (9)

### Hydrogen-bond geometry (Å, °)

**Table:**

*D*—H···*A*	*D*—H	H···*A*	*D*···*A*	*D*—H···*A*
C12—H12···O2^i^	0.93	2.48	3.2675 (17)	143

Symmetry codes: (i) *x*+1, *y*, *z*.
